# Effect of Poly(vinyl alcohol) on Nanoencapsulation of Budesonide in Chitosan Nanoparticles via Ionic Gelation and Its Improved Bioavailability

**DOI:** 10.3390/polym12051101

**Published:** 2020-05-12

**Authors:** Georgia Michailidou, Nina Maria Ainali, Eleftheria Xanthopoulou, Stavroula Nanaki, Margaritis Kostoglou, Emmanuel N. Koukaras, Dimitrios N. Bikiaris

**Affiliations:** 1 Laboratory of Polymer Chemistry and Technology, Department of Chemistry, Aristotle University of Thessaloniki, GR-54124 Thessaloniki, Greece; michailidougeorgia18@gmail.com (G.M.); ainali.nina@gmail.com (N.M.A.); elefthxanthopoulou@gmail.com (E.X.); sgnanaki@chem.auth.gr (S.N.); 2 Laboratory of Inorganic Chemistry and Technology, Department of Chemistry, Aristotle University of Thessaloniki, GR-54124 Thessaloniki, Greece; kostoglu@chem.auth.gr; 3 Laboratory of Quantum and Computational Chemistry, Department of Chemistry, Aristotle University of Thessaloniki, GR-54124 Thessaloniki, Greece; koukarase@chem.auth.gr

**Keywords:** chitosan nanoparticles, sustain release, budesonide, drug release, drug dissolution enhancement, COPD treatment

## Abstract

Chitosan (CS) is a polymer extensively used in drug delivery formulations mainly due to its biocompatibility and low toxicity. In the present study, chitosan was used for nanoencapsulation of a budesonide (BUD) drug via the well-established ionic gelation technique and a slight modification of it, using also poly(vinyl alcohol) (PVA) as a surfactant. Scanning electron microscopy (SEM) micrographs revealed that spherical nanoparticles were successfully prepared with average sizes range between 363 and 543 nm, as were measured by dynamic light scattering (DLS), while zeta potential verified their positive charged surface. X-ray diffraction (XRD) patterns revealed that BUD was encapsulated in crystalline state in nanoparticles but with a lower degree of crystallinity than the neat drug, which was also proven by differential scanning calorimetry (DSC) and melting peak measurements. This could be attributed to interactions that take place between BUD and CS, which were revealed by FTIR and by an extended computational study. An in vitro release study of budesonide showed a slight enhancement in the BUD dissolution profile, compared to the neat drug. However, drug release was substantially increased by introducing PVA during the nanoencapsulation procedure, which is attributed to the higher amorphization of BUD on these nanoparticles. The release curves were analyzed using a diffusion model that allows estimation of BUD diffusivity in the nanoparticles.

## 1. Introduction

Chronic obstructive pulmonary disease (COPD) is characterized by chronic airflow limitation and airways inflammation [1]. It is a progressive life-threatening lung disease that was responsible for the death of 5% of the population globally in the year 2015, according to World Health Organization data. The main causes of the disease are smoking and environmental pollutants. It affects equally men and women and the most susceptible groups among them are mainly smokers and the elderly. The major symptoms of COPD are dyspnea, breathlessness, chronic cough and sputum production. The above-mentioned symptoms consequently provoke serious long-term disability in patients’ everyday routine and possibly cause an early death. Although its serious impact on human life is discernible, to date it remains underdiagnosed. The majority of the patients tend to attribute the symptoms as natural consequences of smoking or the fact that they are ageing and consequently ignore them [2]. 

Treatment of the disease with corticosteroids aims to prevent lung function deterioration, alleviate the symptoms, treat complications as they arise [2] and improve the health status of the patients [3]. Inhaled medication is the keystone of the pharmacological treatment for patients who suffer from COPD [4]. This medication offers distinct therapeutic benefits, namely smaller drug doses, more rapid action and minimization of side effects, while leading a drug to the lungs via the respiratory tract [5]. Typically, corticosteroids are highly effective in anti-inflammatory treatment [6]. 

**Figure 1:**
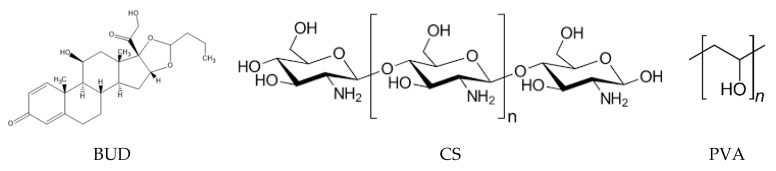
Molecular structures of Budesonide (BUD), chitosan and PVA.

Budesonide (BUD) (Figure 1) is a corticosteroid used in the treatment of asthma, noninfectious rhinitis, COPD [7] as well as in the treatment of chronic gastrointestinal diseases [8]. It is a highly hydrophobic compound with low water solubility and thus low bioavailability in the human body. Recently, many studies have already been conducted aiming to encapsulating BUD using polymers as matrices. Ali et al. encapsulated BUD in PLGA nanoparticles through the emulsion solvent evaporation method [9], whereas Buhecha et al. utilized the double emulsion solvent diffusion technique in order to encapsulate BUD in PLA nanoparticles [10]. Chitosan (CS) is a polymeric carrier used from different research groups aiming to encapsulating BUD. Zhang et al. prepared CS–BUD nanoparticles via a nanospray drying technique [11]. Campos et al. utilized CS with Eudragit in order to form swellable nanoparticles [12] while Bodas et al. constructed CS nanoparticles cross-linked with dextran sulfate through a solvent evaporation method [13]. Eudragit was moreover utilized from Quellini et al. which formulated pH-sensitive BUD loaded nanocapsules through a nanoprecipitation technique achieving controlled release of the drug [14]. Liu et al. used hyaluronic acid in order to prepare microparticles containing BUD through spray dry, aiming in improving the poor solubility of the corticosteroid and ameliorate its pharmacological effect [15] whereas microparticles from poly(vinyl pyrrolidone) were constructed through a supercritical antisolvent method [16]. Furthermore, in order to formulate amorphous powders for inhalation of the drug, there have been efforts to produce spray dried dry powders of BUD with arginine [17] with improved physical stability or with mannitol [18]. Although these groups have already enclosed BUD in various polymeric nanocarriers, the dissolution enhancement of this poorly water-soluble drug (BUD) is not solved and thus new strategies like improved nanoencapsulation procedures are needed and this was examined in the present work. 

Nanoparticles have been widely investigated as suitable candidates for systemic and local administration of a variety of drugs. Their therapeutic nature as drug carriers could proceed either by dissolving, entrapping or encapsulating the active ingredient, or by adsorbing or attaching the active substance [19]. Specifically, pulmonary delivery of nanoparticles has been examined extensively due to enhanced advantages. Pulmonary delivery of nanoparticles has the potential of boosting and sustaining local lung drug concentration for the medication of respiratory disorders. They achieved a shrinkage in dosage frequency which results in enhanced patient compliance and they ensure a sustained release in the lung tissue [20]. However, the size of nanoparticles could be recognized as the most significant determinant that can progress the efficiency of inhaled drug delivery. Several in vitro experiments and mathematical models demonstrate the influence of aerosol particle size to the total lung and regional airway site of inhaled drug deposition [10,11]. In general, smaller particles (300 nm–1000 nm) could attain better total lung deposition in the lower respiratory system and ameliorate airways penetration [21]. 

Chitin [poly (β-(1→4)-N-acetyl-D-glucosamine)] is a natural polysaccharide corresponding to the second most abundant natural polymer after cellulose. It is synthesized by an enormous number of living organisms but commercially it is acquired mostly from the exoskeleton of the crustaceans [22]. Chitosan is the product of chemical or enzymatic deacetylation of chitin [23]. The deacetylation can be partial or complete thus above a 50% deacetylation, the polymer is referred to as chitosan. Therefore, chitosan is composed of deacetylated β(1−4) 2-amino-2-deoxy-β-D-glucan monomers and partially of N-acetyl groups instead of amino groups [24]. After the deacetylation, chitosan becomes a soluble polymer in weak aqueous acid solutions since chitosan’s amino groups are protonated. On the contrary, it remains insoluble in neutral and alkalic environments as well as in many organic solvents. As a polymer, it combines several advantages namely biocompatibility, mucoadhesiveness, biodegradability and non-toxicity [25,26]. Furthermore, it does not provoke allergic reactions and most importantly it is able to self-assemble in acidic aqueous solution and form nanoparticles through ionic cross-linking with polyanions via the ionic gelation technique [24]. The aforementioned technique provides the opportunity to control crucial parameters in the final characteristics of the nanoparticles, for instance size or drug loading efficacy, by alternating the experimental conditions [27]. According to a previous publication from our lab, the size of the formulated nanoparticles depends on the ratio between chitosan and the complexing agent [28]. The formation process of the nanoparticles is very simple and mild. The complexation occurs spontaneously between the positively charged chitosan and the negatively charged polyanion resulting in physically crosslinked due to the electrostatic interactions of the nanoparticles [29]. The encapsulation of active compounds, which are usually molecules of high crystallinity, in chitosan nanoparticles results in their amorphization. Consequently, reducing the crystallinity of the active compounds outcomes in enhancing their bioavailability.

In this study chitosan nanoparticles were prepared via the ionic gelation technique and for the first time, BUD was successfully incorporated in the interior nanoparticle matrix through this method. Furthermore, the addition of PVA during this nanoencapsulation and its effect on BUD dissolution enhancement, was studied for the first time. The properties of the formed nanoparticles and their in vitro release behavior were further studied. The main purpose was the preparation of an effective biocompatible nanocarrier of BUD for pulmonary delivery as well as the amelioration of its in vitro release properties and consequently its bioavailability. For such application, nanoparticles with diameter sizes smaller than 1 μm should be prepared.

## 2. Materials and Methods

### 2.1. Materials and Reagents 

Chitosan with a molecular weight of 18,000 g/mol and a degree of deacetylation > 94%, as was determined by viscometer and ^1^H NMR, respectively [30], was supplied by Kraeber & Co GmbH (Ellerbek, Germany). Sodium tripolyphosphate (TPP) used as ionic crosslinker (85% purity) and acrylonitrile (ACN) were supplied from Aldrich chemicals (Steinheim, Germany). The Budesonide drug (99.99% purity) was kindly donated by Medicair Bioscience S.A. (Athens, Greece). All other materials used were of analytical grade. 

### 2.2. Molecular Weight Determination

The molecular weight of CS was determined by viscometer measurement which was conducted according to Kasaai et al. [31]. Briefly, the intrinsic viscosity [η] was measured at 25 °C using an Ubbelohde viscometer (Type 2C, Witeg Labortechnik GmbH, Wertheim am Main, Germany). CS was dissolved in aqueous solution 0.1 M CH_3_COOH/0.02 M NaCl at final concentration 1% w/v. The intrinsic viscosity [η] of prepared solutions was calculated according to the following equations:

η_r_ = t/t_0_, (1)

η_sp_ = η_r_−1, (2)

η_red_ = η_sp_/c, (3)

[η] = lim_c→0_(η_red_), (4)

where c is the concentration of the solution, t the flow time of solution, and t_0_ the flow time of pure solvent.

The molecular weight was evaluated according to the equation

[η] = 3.04 × 10^−5^M_V_^1.26^. (5)

### 2.3. Nanoparticles’ Preparation

Chitosan nanoparticles were prepared according to a well-established ionotropic gelation technique [32]. In brief, the proper amount of CS was dissolved in 25 mL aqueous solution of acetic acid 2%v/v (pH 4.5), formulating a resulting solution containing 0.8% w/v CS. BUD was dissolved in the minimum required volume of ethanol and was added in CS solution in final concentrations 10 wt%, 20 wt% and 30 wt% in BUD to CS polymer matrix, followed by magnetic stirring for 30 min and probe sonication (100 W, 30 kHz, Hielscher Ultrasonics, Teltow, Germany) for another 2 min. An aqueous solution of TPP, 2mg/mL in concentration and 25 mL in volume, was inserted dropwise to the neat CS solution and to these containing BUD, under magnetic stirring. According to Koukaras et al., there is a crucial ratio between CS and TPP were the nanoparticles are produced with the smallest size [33]. As a result, the ratio of CS/TPP was 4/1. The nanoparticles were stirred for 4 h and centrifuged at 9000 rpm for 20 min. The resulting mixture was left under magnetic stirring for 4 h. By this time, nanoparticles were formed due to interactions taking place between the negative groups of TPP and the positive charged groups of CS. The nanoparticles that were formed were isolated by centrifugation (20 min, 11,000 rpm, Heraeus™ Pico™ 17 Microcentrifuge, Thermo Fisher Scientific, Waltham, Massachusetts, USA.), washed with water and resuspended in water. After the freeze-drier procedure, nanoparticles were kept in a vacuum for further use. 

Nanoparticles were also prepared in the presence of poly(vinyl alcohol) (PVA), used as an emulsifier, in order to enhance BUD solubility. The procedure was the same with the one described above with the addition of CS and TPP, while 2 mL of PVA, 1 wt% in concentration, was added in CS aqueous solution before the addition of BUD solutions. 

### 2.4. Nanoparticle Characterization

#### 2.4.1. Fourier-Transformed Infrared Spectroscopy (FTIR)

FTIR spectra of the samples were obtained using an FTIR spectrometer (model FTIR-2000, Perkin Elmer, Waltham, MA, USA). A small amount of each sample was triturated with a proper amount of potassium bromide (KBr) and the disks were formed under pressure. The spectra were collected in the range of 400 to 4000 cm^−1^ at a resolution of 4 cm^−1^ using 16 co-added scans and the baseline was corrected and converted into absorbance mode.

#### 2.4.2. Wide-Angle X-ray Scattering (XRD)

X-ray powder diffraction (XRD) patterns were recorded using an XRD-diffractometer (Rigaku-Miniiflex II, Chalgrove, Oxford, UK) with a CuKα radiation for crystalline phase identification (λ = 0.15405 nm). The sample was scanned at the range of 5 to 55° with a scan speed of 1°/min.

#### 2.4.3. Dynamic Light Scattering (DLS)

The size of the nanoparticles was determined by dynamic light scattering (Zetasizer 5000, Malvern company, Worcestershire, United Kingdom). Suspended nanoparticles of 100 μL were dispersed in 900 μL of double-distilled water. All measurements were performed in triplicate.

#### 2.4.4. Differential Scanning Calorimetry (DSC)

For differential scanning calorimetry analysis, a Perkin–Elmer Pyris 1 differential scanning calorimeter (DSC) (Waltham, MA, USA) calibrated with Indium and Zinc standards, was used. About 10 mg of each sample was used, placed in sealed aluminum pan and heated up from 30 to 105 °C with a heating rate 20 °C /min in an inert atmosphere (N_2_, flow rate 50 mL/min), held in 125 °C for 10 min in order to remove the absorbed water, cooled to 30 °C with a cooling rate 250 °C/min and heated up again from 30 to 285 °C. The data reported in this work were acquired from the second heating scan.

#### 2.4.5. Thermogravimetric Analysis (TGA)

Thermogravimetric analysis (TGA) was conducted in a Perkin–Elmer Pyris 1 TGA thermogravimetric analyzer (Waltham, MA, USA). Samples of 10 ± 0.5 mg were placed in alumina pans. An empty alumina pan was used as a reference. Heating was conducted from ambient temperature up to 600 °C in a 50 mL/min flow of N_2_. The heating rate was set at 20 °C/min and continuous records of sample temperature, sample weight, and heat flow were recorded. 

#### 2.4.6. Scanning electron Microscopy (SEM)

Scanning electron microscopy (SEM) images were acquired with an electron microscope JEOL 2011 (Akishima, Tokyo, Japan). A drop of each nanoparticles’ suspension was placed to the holder and left to evaporate. Samples were covered with carbon for providing a good conductivity of the electron beam. Operating conditions were set at accelerating voltage 20 kV, probe current 45 nA and counting time 60 s.

#### 2.4.7. High-Pressure Liquid Chromatography (HPLC), Quantitative Analysis and Drug Loading 

Quantitative analysis and drug loading was performed using a Shimadzu HPLC (Kyoto, Japan) prominence system consisting of a degasser (DGU-20A5, Kyoto, Japan), a liquid chromatograph (LC-20 AD, Kyoto, Japan), an autosampler (SIL-20AC, Kyoto, Japan), a UV/Vis detector (SPD-20A, Kyoto, Japan) and a column oven (CTO-20AC, Kyoto, Japan). For the analysis the validated method of Leng et al. was used [34]. In detail, CNW Technologies Athena C18, 120 A, 5 μm, 250 mm × 4.6 mm at a column temperature of 25 °C. The mobile phase consisted of ACN/H_2_O (acidified with phosphoric acid at final pH = 3.2) 60/40 v/v, at a flow rate of 1.0 mL/min. UV detection was performed at 250 nm. The injection volume was 20 μL. The calibration curve was created by diluting a stock methanol solution of 100 ppm BUD to concentrations of 0.01, 0.05, 0.1, 0.25, 0.5, 1.0, 2.5, 5.0 10.0, 20.0 and 30.0 ppm using ultrapure water.

For the determination of the drug loading capacity of the nanoparticles, 10 mg of the prepared nanoparticles was dissolved in 10 mL of aqueous acetic acid solution (1% v/v): methanol (50:50 v/v). The resulting solution was stirred for 24 h, filtered (nylon filters, 0.45 nm pore size). 

#### 2.4.8. Yield, Encapsulation Efficiency and Drug Loading

Nanoparticle yield, drug loading and encapsulation efficiency (EE) were evaluated by applying the following equations:

Yield (%) = [Weight of nanoparticles/Initial weight of polymer and drug] × 100,(6)

Drug loading (%) = [Weight of drug in nanoparticles/Weight of nanoparticles] × 100,(7)

EE (%) = [Weight of drug in nanoparticles/Initial weight of drug] × 100,(8)

#### 2.4.9. In vitro Dissolution Studies

For the in vitro release studies, DISTEK Dissolution Apparatus I (North Brunswick, NJ, USA) equipped with an autosampler was used. Nanoparticles were inserted in dialysis tubing, (molecular weight cut-off 12,000–14,000, Servapor) and placed in the baskets of the apparatus. Dissolution was performed at 37 ± 1 °C and the rotation speed was set at 100 rpm. The dissolution medium was 300 mL of a phosphate buffer, pH = 7.4. Two milliliter of aqueous solution was withdrawn from the release media and quantified according to Leng et al. [34] as was previously described. 

### 2.5. Technical Details on the Computation

Obtaining quantitative and qualitative accurate results and acquiring insight into the interacting systems requires high-accuracy computations from first principles. To this end we performed computations within the framework of density functional theory (DFT) employing judicially chosen functionals, while implicitly accounting for solvent effects with water as solvent. All reported results have been computed using the Gaussian package [35] unless otherwise stated.

#### 2.5.1. Vibrational Analysis

All structures have been optimized employing the gradient corrected B97-D3(BJ) function [36] that includes long-range dispersion corrections handled using Grimme’s D3 dispersion with Beck–Johnson damping [37]. The choice was guided by the requirement to combine accurate molecular geometries as well as configurations that have a dependence on long-range interactions, while maintaining a reasonable computational cost [38,39]. The effects of solvents were accounted for implicitly employing the integral equation formalism variant of the Polarizable Continuum Model (IEFPCM), with water as a solvent [40,41,42,43]. We should note that the meta-GGA variant of the functional, B97M-rV [44], as implemented in the ORCA software package [45], was considered due to its reported exceptional accuracy [39] but its use was discontinued upon encountering erratic performance that we can only attribute to the handling of the kinetic energy term [46]. The triple-ζ basis set of Ahlrichs def2-TZVP [47] was used throughout. Convergence criterium for the SCF energy was set to 10^−7^ Eh, and for the rms of the (one–electron) density matrix up to 10^−8^. Tight convergence criteria were also set for both average and maximum residual forces (norm of the Cartesian gradient) at 1.5 × 10^−5^ a.u., and for both average and maximum residual displacements at 6 × 10^−5^ a.u.

#### 2.5.2. Interaction Energy

At the low pH levels used in this work, the amine groups of chitosan were protonated [24,33]. The maximum interaction energy configuration between Budesonide and CS was modeled using a chitosan pentamer with fully protonated amine groups. All computed points of the potential energy surface scan to establish the interaction energy profile were performed employing the computationally more demanding, range separated ωB97X-D3(BJ) [48], that includes long-range dispersion corrections handled using Grimme’s D3 dispersion with Beck–Johnson damping [37], and that has been reported to provide exceptional performance for non-covalent interactions [39].

## 3. Results and Discussion

### 3.1. Characterization of Budesonide-Loaded Nanoparticles

Nanoparticles were prepared according to the ionic gelation technique. In this technique nanoparticles are formed due to interactions taking place between the negative groups of TPP and the positive charged tertiary amino groups of CS. Analogous studies have already been conducted previously from our group by incorporation of timolol for ocular delivery [28], paliperidone for intranasal delivery [49] and annatto or saffron for the preparation of UV protective cosmetic emulsions [50]. Koukaras et al. [24] studied the effect of the CS:TPP ratio in nanoparticle size formulations. It was found that a ratio of 4:1 resulted in the smallest particles, about 200 nm. Due to the fact that budesonide is mainly used in inhalable systems, the proper ratio was selected in order to eliminate the factor of the size that could affect its delivery through the respiratory tract [51].

**Figure 2:**
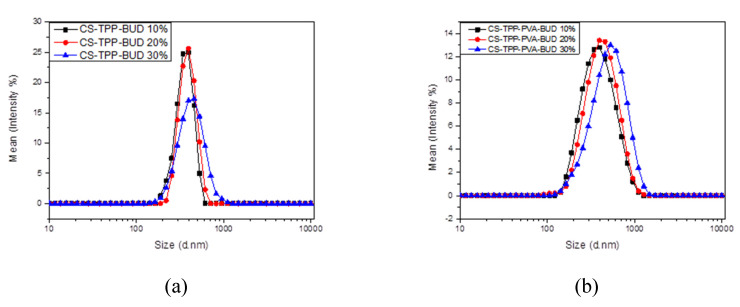
Particle size distribution measured by dynamic light scattering (DLS) of (**a**) CS-TPP-BUD and (**b**) CS-TPP-PVA-BUD nanoparticles for three different BUD concentrations, 10, 20 and 30 wt%.

After the nanoparticles’ formation, washing and isolation steps were implemented as described in the Experimental Section. The prepared nanoparticles were evaluated for their size and zeta potential. The results are shown in Table 1. As can be seen in all formulations, the size varied between 363 and 543 nm showing two specific tendencies; (i) sizes increased proportionally to BUD content and (ii) using PVA during preparation led to nanoparticles with slightly larger sizes. The first trend can be explained by the effect of drug concentration; higher concentration leads to an increase of drug entrapment, which in turn results in the formation of larger nanoparticles. In addition, the addition of BUD could hinder the interactions between TPP and CS groups, leading to larger nanoparticles. The second one is related to the increase in BUD solubility using PVA. This also led to an increase in the amount of drug that could be entrapped into the matrix during its formation leading to larger nanoparticles. In addition, even though PVA is used as a surfactant it is possible that it enters inside the nanoparticles and its dispersion could also adversely affect the ionic interactions, diverging from levels appropriate for the formation of nanoparticles. Similar results have been found in our previous work using CS derivatives with poly(ethylene glycol), which, due to the reduced ionic interactions between CS and TPP, prepared nanoparticles with higher sizes. [52]. Therefore, it is clear that the effect of using PVA is to increase nanoparticle sizes. Another effect of PVA usage can be seen in Figure 2 concerning size distribution. As can be seen, nanoparticles prepared without the presence of PVA exhibit a relatively narrow size distribution curve with larger polydispersity indices (PdI). On the contrary, nanoparticles prepared using PVA display a wider distribution curve, nevertheless they demonstrate ameliorated polydispersity. In any case, the size of the prepared nanoparticles is satisfying for inhalational drug administration. Microparticles larger than 5 μm are generally deposited in the oropharynx region [53] while microparticles 1–5 μm are deposited in the upper respiratory system. Consequently, the required nanoparticle size for an effective release in the lower respiratory area is larger than 300 nm and smaller than 1 μm [21]. 

Zeta potential of nanoparticles is a factor corresponding to surface charge of the nanoparticles in the solution [54] affecting its colloidal stability. Nanoparticles with zeta potential lower than –25 mV and greater than +25 mV are considered as stable colloidal systems and will prevent aggregation [55]. BUD is a pharmaceutical compound with neutral charge and consequently, the zeta potential values shown in Table 1 are attributed to the presence of the polysaccharide. As can be seen, the zeta potential of all nanoparticles is above +25 mV indicating that all formed nanoparticles could exhibit high stability. Interestingly, nanoparticles prepared in the presence of PVA showed slightly higher zeta potential values, which may be attributed to the addition of PVA, which acts as a surfactant preventing nanoparticle aggregation [56]. 

**Figure 3:**
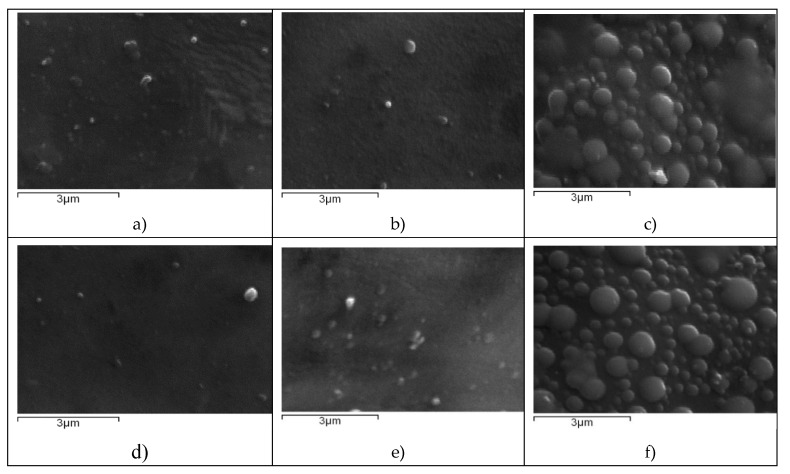
SEM image of: (**a**) CS-TPP-BUD 10%, (**b**) CS-TPP-BUD 20%, (**c**) CS-TPP-BUD 30%, (**d**) CS-TPP-PVA-BUD 10%, (**e**) CS-TPP-PVA-BUD 20% and (**f**) CS-TPP-PVA-BUD 30%.

SEM was used in order to examine the morphology of the prepared nanoparticles. As can be seen in Figure 3a–f, all nanoparticles were spherical in morphology with a smooth surface. SEM photos also revealed the polydispersity of the samples as was also shown by DLS measurements (Table 1). 

**Figure 4:**
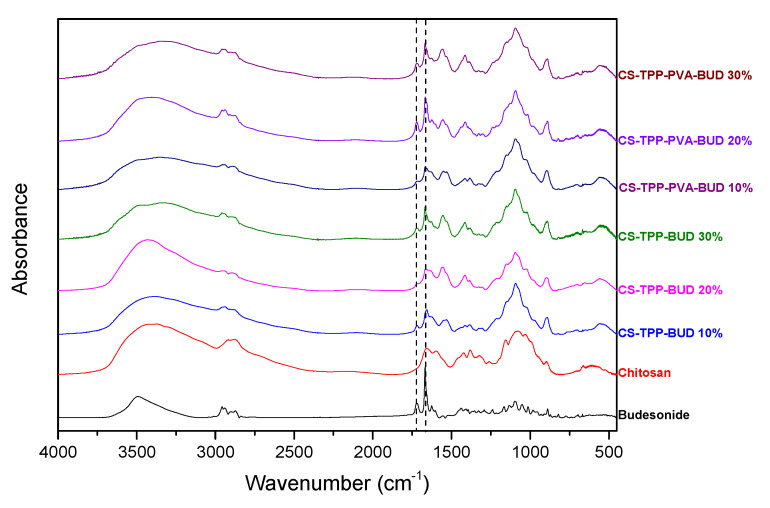
FTIR spectra of CS nanoparticles containing BUD in different ratios 10%, 20%, 30% w/w.

FTIR analysis was used in order to examine ionic interactions between CS and TPP as well as possible hydrogen bond formation between CS and BUD in nanoparticles (Figure 4). CS showed all its characteristic peaks; a broad characteristic region at 3400 cm^−1^ where O–H and N–H stretch bonds peaks were overlapped, vibrations of the amides and primary amines at 1655 and 1596 cm^−1^ respectively, the peak of axial deformation of the C–N bond at 1324 cm^−1^ and finally the peaks at 1074 and 1026 cm^−1^ attributed to C–O bond of cyclic alcohols and to primary hydroxyl groups of the polysaccharide. BUD characteristic peaks appeared at 3491 cm^−1^ corresponding to the vibration of the hydroxylic groups, at 2951 cm^−1^ corresponding to methyl groups vibration (CH_2_), at 1729 and 1667 cm^−1^ and characteristic stretching vibration of carbonyl groups (C=O) and at 1626 cm^−1^ due to stretching vibration of the double bond C=C. According to Papadimitriou et al. [32], at the spectrum of neat chitosan nanoparticles prepared with TPP by ionic gelation, a shift was expected in the characteristic absorbance peaks of the amides of both types as well as the phosphate groups of TPP, which have an absorbance at 897 cm^−1^. As can be seen in CS/TPP nanoparticles this characteristic peak was shifted to lower values (893 cm^−1^), which is evidence of evolved interactions of the amino groups of CS with the phosphate groups of TPP. In BUD unencapsulated nanoparticles, there are also some interesting findings. Neat BUD has characteristic absorbances at 3491 cm^−1^ due to its –OH groups, two carbonyl stretching bands at 1729 cm^−1^ and at 1667 cm^−1^ attributed to the nonconjugated acetyl C=O stretch and conjugated dihydrobenzoquinone C=O groups, respectively. In BUD encapsulated nanoparticles it is not possible to detect any differentiation to –OH absorbance due to the broad peak of CS hydroxyls in all nanoparticles. However, there is a small shift of the first >CO peak absorption from 1729 cm^−1^ to 1725 cm^−1^, while the other one 1667 cm^−1^ remains almost stable (1665 cm−1). Looking more carefully in this area a small peak was evident in all nanoparticles as a shoulder at 1655 cm^−1^, which was not present in neat BUD. These shifts and new absorbances could result from interactions of these groups with amino or hydroxyl groups of CS, probably via hydrogen bonding. Similar interactions have been reported in the literature between BUD and poly(ethylene glycol) [57].

**Figure 5:**
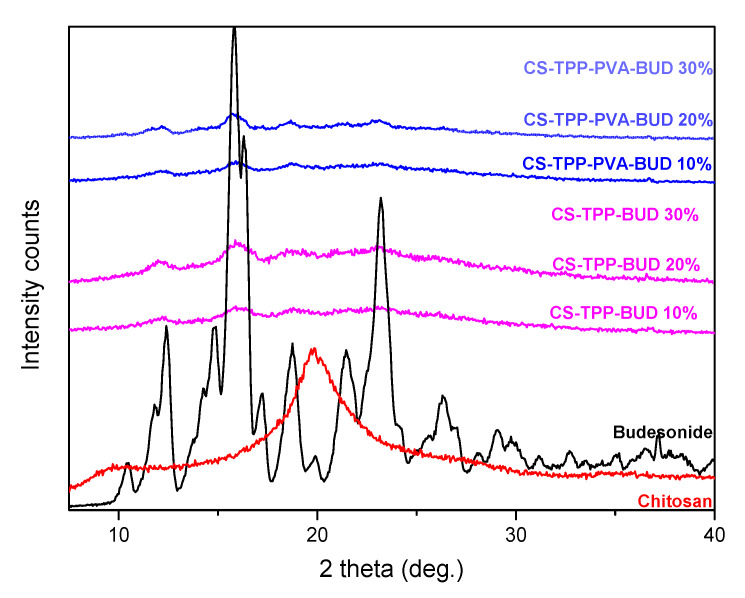
XRD patterns of CS, BUD and CS nanoparticles containing BUD in different concentrations of 10%, 20%, 30% w/w.

X-ray diffraction analysis was conducted in order to assess the physical state of BUD in chitosan nanoparticles. Chitosan is a semi-crystalline polymeric material with two characteristic peaks at 11° and 21° [50]. In contrast, BUD is a crystalline pharmaceutical compound with a sharp peak at 15.8°, secondary peaks at 12.2°, 23.2° and many smaller peaks as well [9]. XRD patterns of the CS nanoparticles prepared showed that BUD is incorporated in its crystalline form (Figure 5). In brief, in all prepared formulations, the sharper peaks of BUD are present at 12.2°, 15.8° and 23.2° while that of CS were absent, maybe because the recorded patterns in all nanoparticles have been broadening. A decrease in the crystalline phase of CS was also observed which is attributed to the rearrange of intermolecular and intramolecular network of the polymer, due to the crosslinking with the TPP ions. The peak at 21^o^ is not recorded as in neat CS. Furthermore, the intensity of the characteristic peaks of BUD is drastically reduced in all nanoparticles while it is clear that at low drug content like 10 wt%, the peak intensities are too small. In the other nanoparticles the peak intensities are progressively increased by increasing drug content. Comparing the peak intensities of nanoparticles prepared without PVA and with PVA it is clear that these are slightly higher in nanoparticles without emulsifier. From these patterns and peak intensities, as well as peak areas, the degree of crystallinity of encapsulated BUD was calculated. For CS/TPP/BUD nanoparticles containing 10 wt%, 20 wt% and 30 wt% BUD, a high degree of crystallinity like 77%, 94% and about 97% was calculated, respectively. These values are much reduced for CS/TPP/BUD/PVA and for nanoparticles containing 10 wt%, 20 wt% and 30 wt% BUD, a degree of crystallinity of about 55%, 68% and 82%, was calculated, respectively. Therefore, it is clear that PVA really plays the role of an emulsifier and reduces the ability of BUD to be crystallized in nanoparticles. 

**Figure 6:**
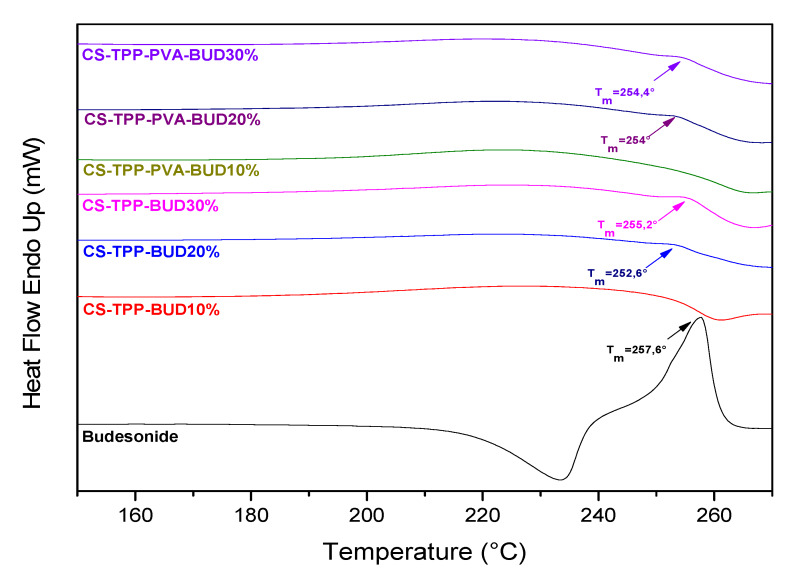
DSC curves of chitosan nanoparticles containing BUD.

DSC measurements were also performed in order to evaluate the physical state of BUD in chitosan nanoparticles, as well as the evolved interactions between them. Figure 6 shows thermographs of the pure drug as well as the encapsulated nanoparticles. BUD is a crystalline compound which, as reported by the literature, has a characteristic endothermic melting peak at 257 °C [34]. 

Moreover, chitosan’s thermogram does not show any significant transition along the range of temperatures 50–270°C [58,59]. Consequently, the endothermic melting peaks are attributed only to the presence of BUD. Nanoparticles containing 10% of the drug with or without the presence of PVA did not show any T_m_ value due to its low content or due to a higher amorphization percentage of BUD. In contrast, samples containing 20% and 30% of BUD appear to have very small T_m_ peaks, indicating the presence of drugs in a crystalline structure. More precisely, the samples CS-TPP-BUD-20% and CS-TPP-BUD-30% showed T_m_ at 252.6 °C and 255.2 °C respectively, whereas the samples CS-TPP-PVA-BUD-20% and CS-TPP-PVA-BUD-30% showed melting peaks at 254 °C and 254.4 °C respectively. This slight reduction in melting point may be explained by the interaction between BUD and CS, which led to a broadening of the melting peak of budesonide as well as a shift towards lower temperature. This could also be attributed to the reduction of the degree of crystallinity on encapsulated BUD. DSC results are in agreement with the results derived from XRD measurements.

**Figure 7:**
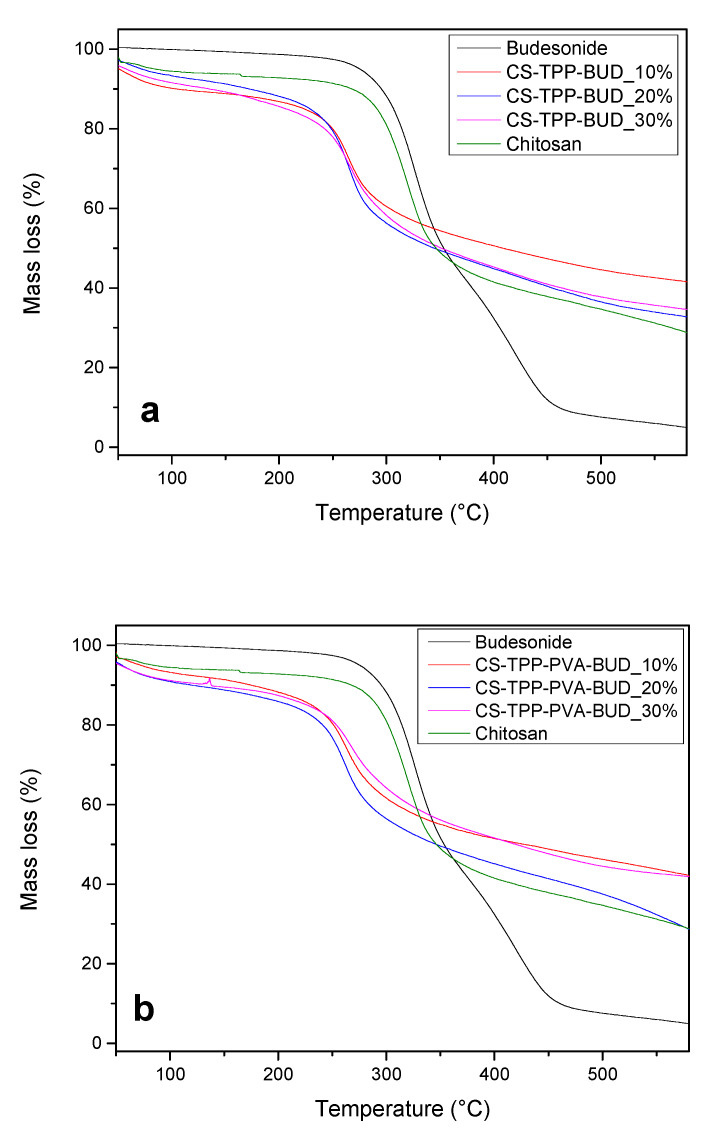
TGA thermograms of CS, BUD and chitosan nanoparticles containing BUD (**a**) without emulsifier and (**b**) with emulsifier.

Thermogravimetric analysis was conducted in order to assess the weight loss of the fabricated nanoparticles during heating cycles and their stability during storage. Figure 7a,b shows the thermographs of pure chitosan, BUD and of the formulated nanoparticles. More specifically, pure BUD demonstrates a one-step mass loss between 250–370 °C while at 600 °C the mass residue is about 5%. In contrast, the thermogram of CS has two main decomposition steps. The first step at 50–100 °C occurs due to the evaporation of the unbound water while the second at 250–400 °C is attributed to the dehydration and decomposition of chitosan’s polymeric chains [60]. Interesting is the fact that the mass loss thermograms of the nanoparticles follow the two-step thermal degradation of CS. However, a shift is observed of the second mass-loss degradation step of nanoparticles at lower temperatures in comparison to neat CS and BUD. This shift is attributed to the interaction between the drug and the polymeric matrix as well as disorders in the crystalline structure of the nanoparticles. The same effect is described by Kahdestani et al. while studying the encapsulation of teicoplanin in CS nanoparticles [61]. Therefore, from these thermograms it is not possible to see the effect of BUD in nanoparticles. However, it is clear that the prepared nanoparticles should be stable during storage.

### 3.2. Computational Study

#### 3.2.1. Intermolecular Interactions

**Figure 8:**
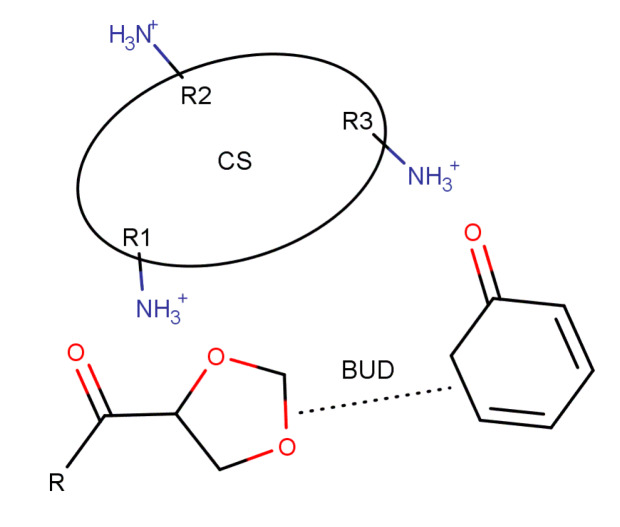
The configuration with the strongest interaction between Chitosan and Budesonide has two sites, one for each carbonyl oxygen atom of BUD. Each carbonyl oxygen interacts with the second nearest protonated amino group.

From the characterization of encapsulated BUD nanoparticles and mainly from FTIR, XRD and DSC it was found that some interactions are taking place between drug and CS. However, it is very difficult to evaluate the extent of these interactions. Only a few pieces of evidence have been revealed and for this reason we procced in a computational study to identify these interactions more precisely and uncover the underlying mechanism. At the maximum interaction configuration oxygen atoms of BUD interact non-covalently with hydrogens of protonated amino groups ($-{\mathrm{NH}}_{3}^{+}$) of CS at two sites. The size of BUD is such that the configuration allows for the two carbonyl groups to interact with (the amino groups of) two nearest non-consecutive CS rings. Furthermore, at one of the interaction sites (not involving the cyclohexadienone group), in addition to the carbonyl oxygen, the nearby oxygen of the dioxolane group also interacts with the same CS amino group. The configuration is outlined in Figure 8. 

**Figure 9:**
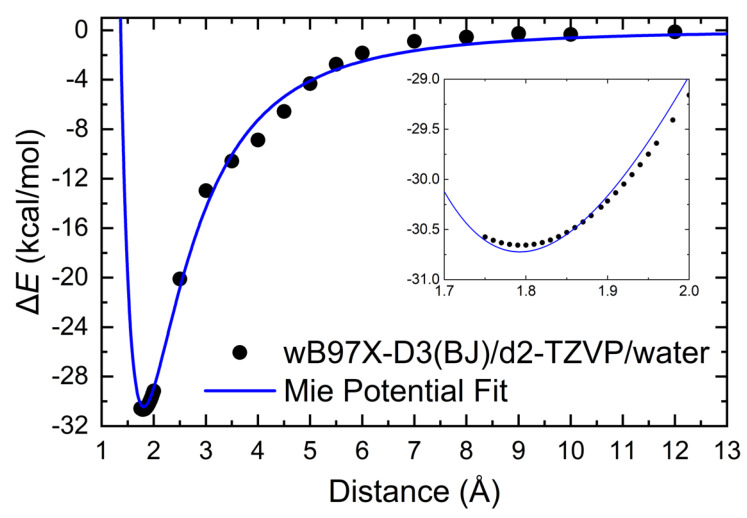
Interaction energy of BUD with CS that has fully protonated amino groups. The inset enlarges the region at the bottom of the energy well. The blue line is a fit of the computed data points to a Mie potential (see main text). Datapoints (solid circles) were computed at the ωB97X-D3(BJ)/def2-TZVP/PCM level of theory.

The interaction profile shown in Figure 9 was computed by scanning CS–BUD separation and by measuring the distance between the oxygen atom of the dioxolane group of BUD and the nearest hydrogen atom of the interacting amino group of CS. The relative orientation of BUD and CS is such that the increase in distance lies nearly on the plane of the interacting (and the neighboring) CS monomer.

The interaction energy $E_{\mathrm{int}}$ was calculated at each point of the potential energy surface (PES) scan, as

(9) $$E_{\mathrm{int}}=E_{\mathrm{BUD}+\mathrm{CS}}\;–\;\left(E_{\mathrm{BUD}}+E_{\mathrm{CS}}\right),$$

where $E_{\mathrm{BUD}+\mathrm{CS}}$ is the energy of CS and BUD interacting system, $E_{\mathrm{BUD}}$ is the energy of the Budesonide molecule, and $E_{\mathrm{CS}}$ is the energy of a fully protonated CS pentamer, i.e., that carries a charge of +5e. The depth of the potential well is computed at 30.7 kcal/mol, indicative of the significant strength of the overall (combined) interaction.

The profile of the interaction has been fitted to a Mie potential, U, that has the general form

(10) $$U\left(r\right)=\frac{n}{n-m}{\left(\frac{n}{m}\right)}^{\frac{m}{n-m}}\epsilon \;\left[{\left(\frac{\sigma }{r}\right)}^{n}\;–\;{\left(\frac{\sigma }{r}\right)}^{m}\right],$$

where r is the distance between CS and BUD, ϵ is the depth of the energy well, and σ is the distance $r_{0}$ for which $U\left(r_{0}\right)=0$ (and the minimum located at $r_{\min}={\left(\frac{n}{m}\sigma^{\left(n-m\right)}\right)}^{\frac{1}{\left(n-m\right)}}$). Fitting of the computed data points results in ϵ=30.7 kcal/mol, σ=1.35 Å, n=4.19, m=2.93.

#### 3.2.2. Infrared Spectra

**Figure 10:**
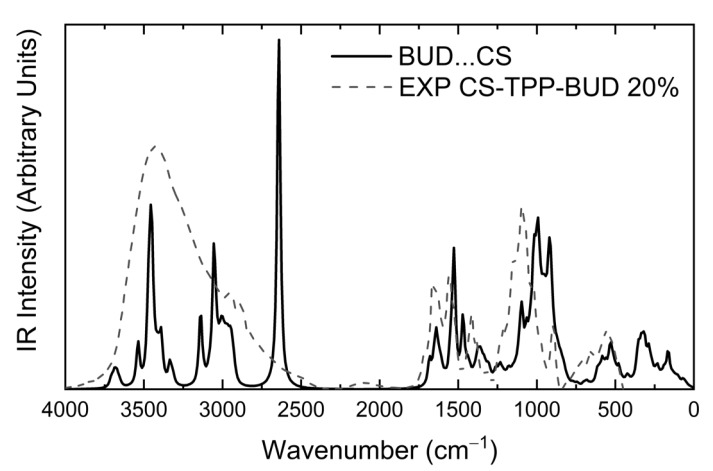
Experimental (FT-IR) and computed infrared spectra of interacting Chitosan–Budesonide system. The experimental curve (dashed line) corresponds to CS nanoparticles containing 20% w/w BUD. The theoretical curve (solid line) was computed at the B97-D3(BJ)/def2-TZVP/PCM level of theory.

Information on the encapsulation of Budesonide by Chitosan nanoparticles and their interactions can be extracted by examining the IR spectra combined with vibrational analysis on the interacting components. To this end, in Figure 10 we have plotted the experimental spectrum that corresponds to the case of CS nanoparticles containing BUD at a ratio of 20% w/w, alongside the computed infrared spectrum for BUD interacting with a protonated CS pentamer. The computed IR spectrum was obtained at the B97-D3(BJ)/def2-TZVP/PCM level of theory with water as solvent. CS is taken fully protonated (accounting for protonation of CS amino groups at working pH levels) [24,33]. Gaussian broadening with a half-width at half-height of 12 cm^−1^ was applied to the computed eigenvalues to produce the simulated curves. A direct comparison between computed and experimental spectra reveals different trends of the computed spectra at large and small wavenumbers. At large wavenumbers the computed values are overestimated by about 90 cm^−1^, and at smaller wavenumbers underestimated by about the same amount. We first turn our attention to the large wavenumber region. Compared to the computed IR spectrum of CS (provided in the Supporting Information in Figure S2), the spectrum of the CS–BUD interacting system exhibits wider bands in the range 2750 cm^−1^–3750 cm^−1^, and with a fine structure (several subpeaks). The relative overall heights of the bands are more levelled in the composite system, whereas for pure CS the band at 3000 cm^−1^ is more intense. This is reflected in the experimental spectra, shown in Figure 10, as well as in Figure 4. With the exception of the samples with a 20% w/w CS concentration, the band at 2900 cm^−1^ is of comparable height to the band centered at 3400 cm^−1^. In the cases with a 20% w/w CS concentration, the band at 3420 cm^−1^ stands out in comparison. This is in agreement with the assessment that these samples exhibit higher drug loading efficiency and will be discussed in the following (Table 2). We further examine this via Figure S1a–c of the Supporting Information that shows a decomposition of the experimental spectra into gaussian subpeaks. The decomposition clearly shows that the 10% w/w and 30% w/w CS concentration samples both have contributions above 2600 cm^−1^ from an intense broad band, whereas the 20% sample has minimal contributions up to 3000 cm^−1^. This effectively results in the noted difference in heights for the 20% sample and is a signature of the higher BUD content (due to interaction and not introduction of pure BUD vibrational states). At this point we should note that the intense peak of the composite system at 2600 cm^−1^ emerges from a single state of a stretching N–H mode of a protonated amine group that is interacting with a BUD C=O carbonyl group. This mode is present as a low intensity shoulder in all of the experimental of Figure 4, located at 2500 cm^−1^–2520 cm^−1^.

The remaining peaks of interest are located at the small wavenumber range. The most interesting difference at this range between the infrared spectra of pure CS and that of the CS–BUD interacting system is located around 1500 cm^−1^. The amino group wagging modes of protonated CS are identified in the region of 1466–1515 cm^−1^. The amino group wagging modes for the composite system are found in the range of 1469–1472 cm^−1^ when the amino group is not interacting with oxygen atoms of BUD, whereas the modes are shifted by about 70 cm^−1^ to the rage 1536–1550 cm^−1^ for amino groups interacting with carbonyl oxygens of BUD. This is reflected in the experimental infrared spectra (see CS-TPP-BUD spectra of Figure 4), specifically, for CS, a shoulder located at 1460 cm^−1^ of the local band is absent in the spectra of the composite system, for which system, however, a nearby band is present in the range between 1500 cm^−1^ and 1580 cm^−1^. The lack of this shoulder and the intensification of the nearby band is a signature of BUD interacting with CS. It should be noted that at the low end (and slightly below) of the specific spectral region, i.e., below 1480 cm^−1^ (of the computed spectrum), lie the CH_2_ scissoring modes that contribute to the spectrum down to 1421 cm^−1^. A noteworthy intense mode located at 1526 cm^−1^ corresponds to the C=O stretching mode of the cyclohexadienone group (while interacting with a CS amino group).

A peak of interest is located on the far end of this region at about 1680 cm^−1^. The peak, which also manifests in the experimental spectrum of the composite system, corresponds to the C=O stretching mode (not of the cyclohexadienone group) at 1684 cm^−1^ combined with the stretching mode of the interacting protonated amino groups at 1676 cm^−1^. However, the same C=O mode is also identified in the IR spectrum of BUD (see Supporting Information) at 1680 cm^−1^, so it cannot be effectively used to identify BUD–CS interaction but does allow for identification of the presence of BUD. We note that the C=O stretching mode of the cyclohexadienone group in the IR spectrum of BUD is located nearby at 1565 cm^−1^. Additional characteristic modes are the C–O_2_ symmetric stretching mode of the dioxolane group of BUD is located at 1064 cm^−1^; and the wagging modes of non-interacting amino groups are noted in the range of 1169–1150 cm^−1^, and at 1130–1137 cm^−1^ for interacting amino groups. All the above reveal that the main interactions between CS and BUD drugs are between amino groups of CS and carbonyl groups of BUD, which have been also found from FTIR measurements (Figure 4). 

### 3.3. Drug Release Data Analysis

The entrapped efficiency of BUD in the interior of chitosan particles is one of the parameters of the formulated nanoparticles that affects the release behavior leading to an ameliorated bioavailability [62]. Table 2 summarizes yield (%), drug loading efficacy (%) and encapsulation efficiency (%) of all the prepared nanoparticles. It was observed that the yield of the nanoparticles was increased in accordance with the concentration of the drug used. This monotone (near-proportional) dependence was not observed in drug loading results in which the 20% formulations showed the highest values. The hydrophobic nature of BUD has an impact on its loading efficacy to the nanoparticles’ interior. Hydrophobic interactions take place between BUD molecules and consequently, 30% formulations resulted in lower encapsulation efficacy. On the contrary, the formulation prepared by the presence of PVA showed an increase in all drug concentrations, which is an indication that PVA promotes drug encapsulation and the BUD amount incorporated in nanoparticles. 

**Figure 11:**
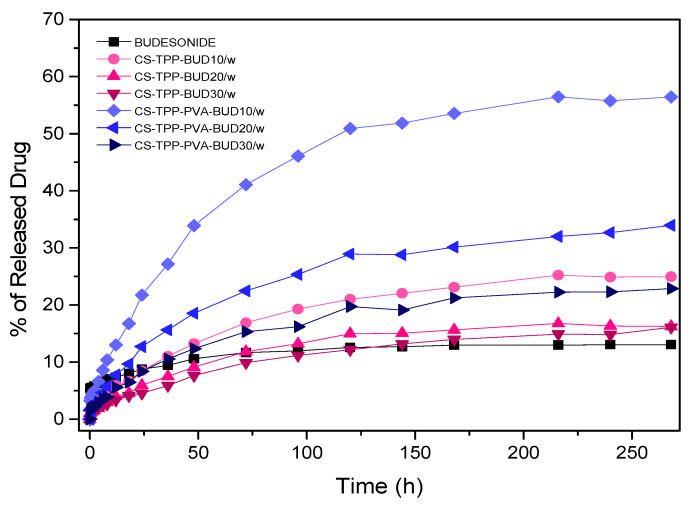
In vitro release of BUD from CS nanoparticles at pH 7.4.

A dissolution study was also conducted. Figure 11 shows the dissolution profiles of pure BUD and its formulations. As has already been mentioned, BUD is a hydrophobic drug and its dissolution percentage showed to be only 13% after 11 days. Concerning the release of BUD from the nanoparticles, it was revealed that the encapsulation of the drug in chitosan nanoparticles results in drug release improvement. Interesting is the fact that while the percentage of the drug increases, the release rate is decreasing. This is attributed to the hydrophobic nature of BUD, but also to its crystalline phase in nanoparticles. Similar findings have been reported in the literature when poorly water-soluble drugs are embedded in polymeric matrices and when drug amount increase inside the polymer matrices, drug release decreases [63,64,65,66]. However, as can be seen only in nanoparticles with 10 wt% BUD there is a small dissolution rate enhancement, while for the other BUD concentrations the enhancement is too small. In brief, in formulations for which no PVA was used the release rate of CS-TPP-BUD 20% and 30% resemble the release of the pure drug without vastly affecting the final release percentage, whereas the sample CS-TPP-BUD 10% is capable of releasing only 23% of the encapsulated drug within 11 days. This is due to the high degree of crystallinity that BUD has in CS/TPP/BUD nanoparticles containing 20 wt% and 30 wt%, which as was found by XRD is about 94% and about 97%, respectively. In contrast, samples where the PVA emulsifier is present revealed an improved dissolution profile. In agreement with the former nanoparticles, the sample which exhibits the best in vitro behavior is the CS-TPP-PVA-BUD 10%, which released up to 56% of the encapsulated drug in a period of 11 days. The release rate is higher in CS/TPP/BUD/PVA nanoparticles with 20 wt% BUD and slightly reduced to 30 wt%. This is because in corresponding nanoparticles BUD was encapsulated in a lower degree of crystallinity, such as 68% and 82%, respectively. This improvement in dissolution rates should be attributed to the higher amorphous drug percentage inside the nanoparticles. It is well known that amorphous drugs have about 1000 higher solubility than their crystalline structures [67,68]. In addition, this is due to the role of PVA as an emulsifier. Similar dissolution enhancements have been found in other poorly water soluble drugs like felodipine and nimodipine, using poly(ethylene glycol) as an emulsifier [65,69]. Consequently, results imply that the presence of the emulsifier affects the release of BUD from the interior of the nanoparticles, as it prevents aggregation and moreover they clearly indicate that the proposed chitosan nanoparticles have a potent ability to improve the in vitro release of BUD. 

**Figure 12:**
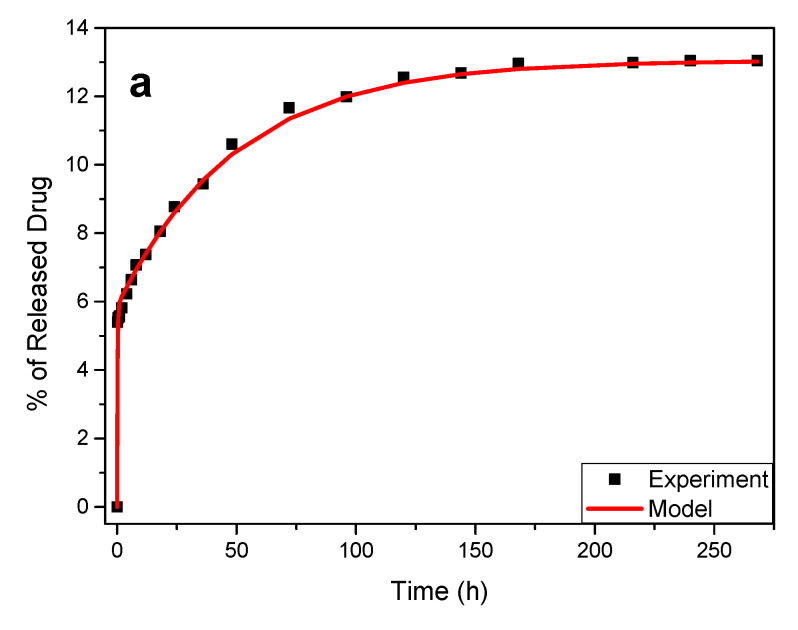

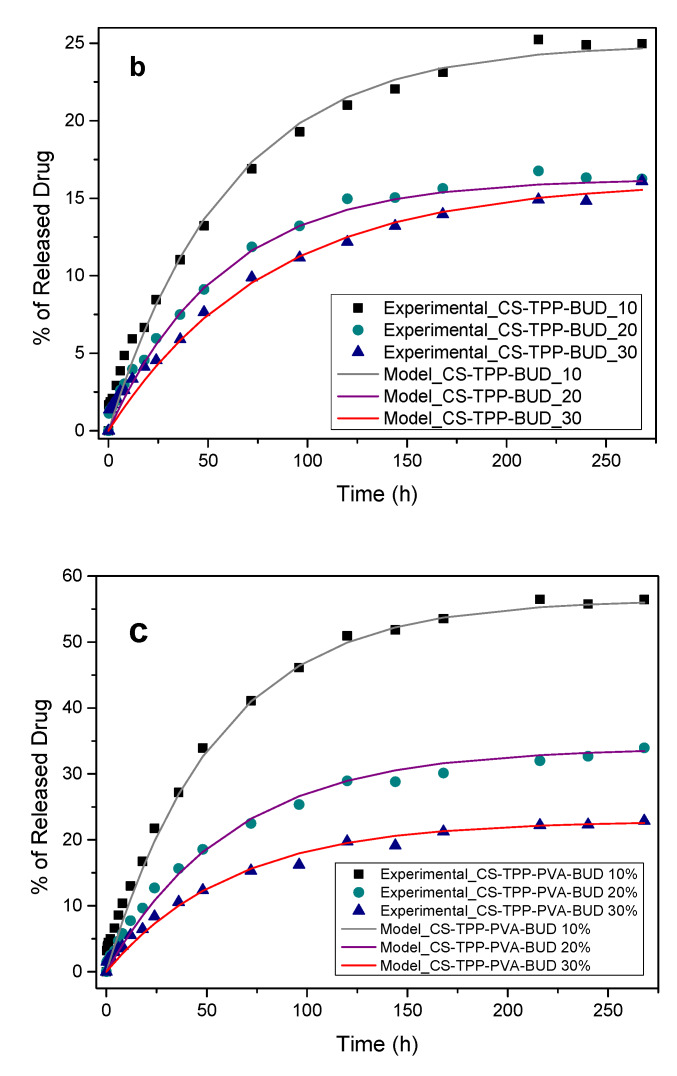
Comparison between experimental (symbols) and model (continuous lines) drug release data for (**a**) neat BUD (**b**) CS-TPP-BUD (**c**) CS-TPP-PVA-BUD.

In this section, an attempt was also made to understand and analyze the drug release mechanism. The first step is the analysis of the pure drug dissolution. The final steady-state is due to the fact that the bulk concentration of the drug has reached the solubility limit. A simple mass balance VC_eq_ = R_f_M/100 (where R_f_ is the final percentage dissolution of the pure drug, V is the liquid volume and M is the drug mass) leads to the estimation of drug solubility in the buffer as C_eq_ = 5 mg/L. Traditional dissolution kinetic mechanisms like the Noyes–Whitney equation [70] cannot describe the experimental dissolution curve. Instead it was found that a double exponential function can perfectly fit the experimental data as can be seen in Figure 12a. The form of the curve is:

R% = R_f_[φ(1-exp(-k_1_t)) + (1-φ)(1-exp(-k_2_t))], (11)

where t is the time, φ is the fraction of the dissolved drug having undergone dissolution with kinetic constant k_1_ and 1-φ is the fraction having undergone dissolution with kinetic constant k_2_. The fitting parameters are: R_f_ = 13.05, φ = 0.45, k_1_ = 5 hr^−1^ and k_2_ = 0.02 hr^−1^. This means that almost half of the drug in the bulk has been dissolved with each mechanism. There is a tremendous difference in the kinetics of the two mechanisms (the first is 250 times faster than the second). The characteristic time of the first mechanism is 12 min so the mass transfer to the bulk may have some contribution to its kinetics. The second mechanism has a characteristic time of 50 h so it is clear that no mass transfer contribution is possible.

In the following, the release from the nanoparticles will be analyzed. The dominant mechanism is the diffusion of the drug through the polymer matrix. Fickian diffusion is assumed as a first approach. In order to avoid repetition of the nanoparticles composition, the six types of nanoparticles CS-TPP-BUD10, CS-TPP-BUD20, CS-TPP-BUD30, CS-TPP-PVA-BUD10, CS-TPP-PVA-BUD20, CS-TPP-PVA-BUD30 are renamed as cases A1, A2, A3, B1, B2, B3 respectively.

The reason for release completion needs to be examined first. Performing detailed mass balances for the drug using the data from Table 2, it is found that the drug concentration in the liquid at the end of the release process is 0.615, 1.192, 1.155, 1.2, 2.82, 1.02 mg/L for cases A1, A2, A3, B1, B2, B3 respectively. It is clear that the drug concentration is significantly below the solubility limit estimated above so the mechanism of release completion is the inability of the rest of the drug to diffuse in the nanoparticle. The reason for this is probably some degree of crystallinity as it has been discussed before. This view is further supported by the fact that the percentage of trapped drug in the particles increases as the initial loading of drug in the particle increases (higher loading facilitates crystallization). The drug loading for A-type nanoparticles is 1.54%, 3.9%, 4.68% and the corresponding trapped percentages are 75%, 83.75%, 83.9%. The relevant values for B type nanoparticles are 1.77%, 4.04%, 5.16% and 43.56%, 66.1%, 77.1% respectively.

The kinetic modelling task is complicated from the fact that there is a particle size spectrum instead of a single particle size. Therefore, the diffusion partial differential equation must be solved for each particle size to compute the drug release rate [71]. In order to simplify the problem, an approximate solution of this equation based on a polynomial assumption for the intraparticle drug loading profile (i.e., linear driving force formula-LDF [72]) will be employed. The large characteristic time of the release process allows us to neglect mass transfer to the bulk contribution so the LDF equation takes the form:

dq/dt = (15D/r^2^)(q_f_ - q), (12)

where q is the instantaneous drug loading during the release process, D is the diffusion coefficient of the drug in the polymer matrix, q_f_ is the final drug loading and r is the nanoparticle radius. The major deviation of the approximating Equation (12) from the solution of the complete diffusion equation, is its inability to capture the initial burst. However, since the present release data do not show appreciable initial burst the use of the simplified Equation (12) is quite reasonable. It is evident that the release rate is not uniform but depends strongly on particle size. The simplest approach to overcome this complication is to assume that all the particles are similar, having a radius equal to the average radius of the size distribution (i.e., the so-called monodisperse approximation). After some algebra the equation is transformed to the following relation for the release curve:

R% = R_f_ (1 − exp (-60Dt/d_av_^2^)), (13)

where d_av_ is the number average nanoparticle diameter which can be found in Table 1. The above theoretical expression is fitted to the experimental release data through a least square minimization procedure. The comparison between experimental and theoretical data appears in Figure 12b,c.

The values of the diffusion coefficient resulting from the fitting procedure are 1 × 10^−20^, 1.25 × 10^−20^, 1.13 × 10^−20^ m^2^/s for the cases A1, A2, A3 respectively. These values are very small as is expected for the diffusion of a drug in a polymer matrix. It can be argued that for CS-TPP-BUD nanoparticles the diffusion coefficient of BUD is independent from the BUD loading. The diffusion coefficients for CS-TPP-PVA-BUD nanoparticles are 1.44 × 10^−20^, 1.48 × 10^−20^, 2.18 × 10^−20^ m^2^/s for the cases B1, B2, B3 respectively. It appears that the presence of PVA in the nanoparticles leads to higher diffusion coefficients. The present model allowed for the characterization of the nanoparticle structure with respect to the BUD diffusivity in it.

## 4. Conclusions

In the present study, BUD loaded CS nanoparticles were successfully prepared via an ionic gelation technique in different ratios and PVA was also used as an emulsifier in order to enhance BUD solubility. SEM images depicted the successful preparation of spherical nanoparticles while DLS measurement showed the actual size of the prepared nanoparticles, which varied from 363 nm to 543 nm. FTIR spectra confirmed that week interactions are taking place between CS and BUD and mainly between amino groups of CS and carbonyl groups of BUD, which was also proved by computational studies. XRD analysis showed that DUD was encapsulated in crystalline state in all nanoparticles, while these also containing PVA have a higher reduced degree of crystallinity. DSC thermograms confirmed XRD results. The TGA thermograms showed that all nanoparticles are thermally stable. Finally, in vitro release studies indicated the enhanced release of BUD in the dissolution medium. However, this is more pronounce in nanoparticles containing PVA as an emulsifier, which promotes the drug dissolution. This is due to the higher drug amorphization in these nanoparticles. As it was found through a mathematical model of the release process, there is a relatively small compositional dependence of the diffusion coefficient of BUD in the nanoparticles. 

## Figures and Tables

**Table 1:** Nanoparticles size, polydispersity index (PdI) and zeta-potential results.

**Sample**	**Z-Average (d.nm)**	**PdI**	**Zeta Potential (mV)**
CS-TPP-BUD 10%	363	0.52	+36.3
CS-TPP-BUD 20%	394	0.60	+37.6
CS-TPP-BUD 30%	443	0.53	+46.9
CS-TPP-PVA-BUD 10%	416	0.26	+38.6
CS-TPP-PVA-BUD 20%	448	0.35	+43.9
CS-TPP-PVA-BUD 30%	543	0.45	+48.7

**Table 2:** BUD drug-loaded nanoparticles yield, drug loading and encapsulation efficiency.

Sample	Yield (%)	Drug Loading (%)	EE (%)
CS-TPP-BUD 10%	38.1	15.4	64.5
CS-TPP-BUD 20%	41.8	19.5	48.8
CS-TPP-BUD 30%	44.2	15.6	29.8
CS-TPP-PVA-BUD 10%	38.2	17.7	74.5
CS-TPP-PVA-BUD 20%	45.1	20.2	54.5
CS-TPP-PVA-BUD 30%	49.3	21.2	36.7

## Supplementary Materials

The following are available online at https://www.mdpi.com/2073-4360/12/5/1101/s1, Figure S1: Decomposition of FTIR spectra at large-wavenumbers for the cases of (a) 10%, (b) 20%, and (c) 30% w/w BUD concentration. The 20% w/w case shows the subpeak at 2900 cm^−1^ with less than half the intensity of the large wavelength band (i.e. centered at ~3400 cm^−1^) and exhibits the highest drug loading efficiency. The experimental curve (solid bold gray line) is decomposed in guassian peaks (dotted lines) that combined form the fitted curve (dashed line), Figure S2: Experimental (FT-IR) and computed infrared spectra of (top) Chitosan, and (bottom) Budesonide. The experimental curves are shown with dashed lines and the theoretical curve with solid lines, computed at the B97-D3(BJ)/def2-TZVP/PCM level of theory.
