# Supplementary material for: Effect of Poly(vinyl alcohol) on Nanoencapsulation of Budesonide in Chitosan Nanoparticles via Ionic Gelation and Its Improved Bioavailability
Source: Polymers (Basel). 2020 May 12;12(5):1101. doi: 10.3390/polym12051101 (PMC7285374; doi:10.3390/polym12051101)
Supplement: Supplementary file 1 [file polymers-12-01101-s001.pdf]

Supporting Information

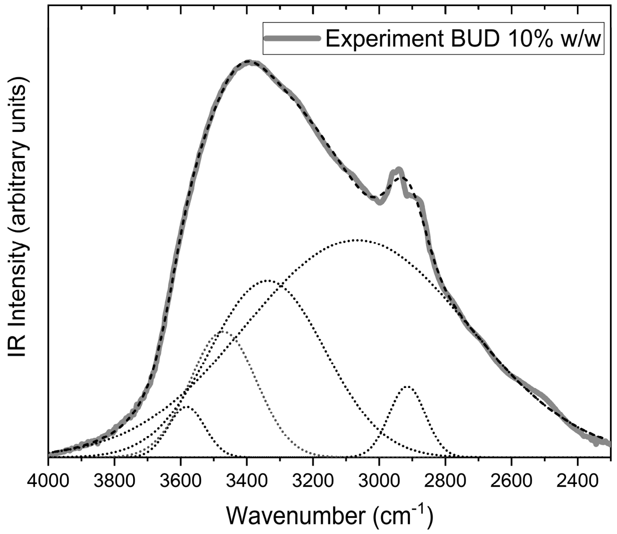

(a)

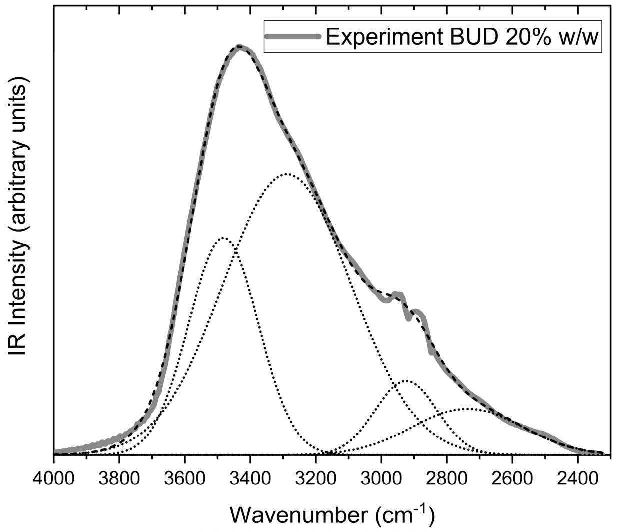

(b)

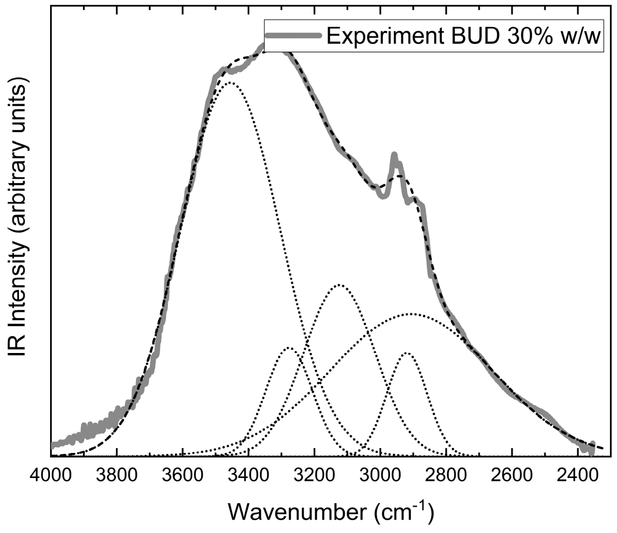

(c)

Figure S1 1a-c. Decomposition of FTIR spectra at large-wavenumbers for the cases of (a) 10%, (b) 20%, and (c) 30% w/w BUD concentration. The 20% w/w case shows the subpeak at 2900 cm^{-1} with less than half the intensity of the large wavelength band (i.e. centered at ~3400 cm^{-1}) and exhibits the highest drug loading efficiency. The experimental curve (solid bold gray line) is decomposed in guassian peaks (dotted lines) that combined form the fitted curve (dashed line).

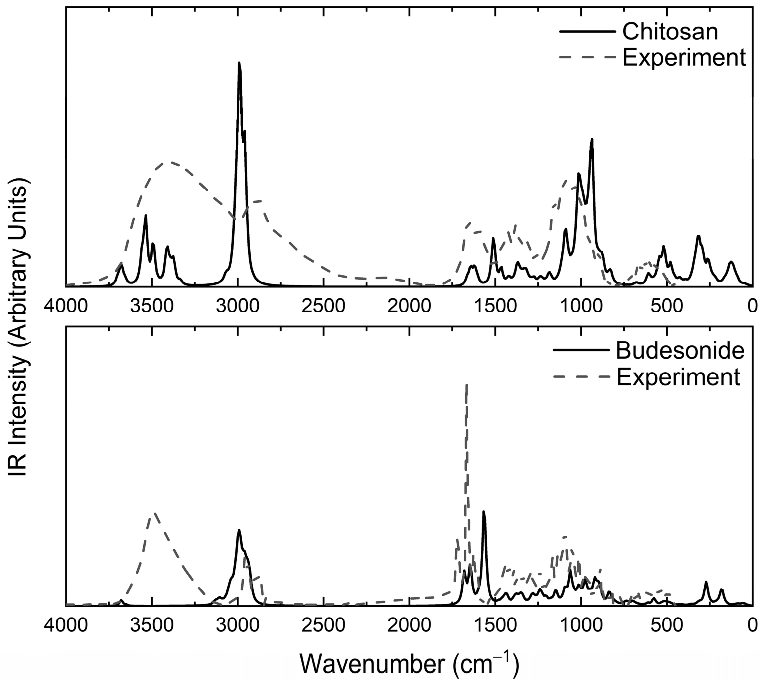

Figure S2. Experimental (FT-IR) and computed infrared spectra of (top) Chitosan, and (bottom) Budesonide. The experimental curves are shown with dashed lines and the theoretical curve with solid lines, computed at the B97-D3(BJ)/def2-TZVP/PCM level of theory.
